# Applying a systems oriented ethical decision making framework to mitigating social and structural determinants of health

**DOI:** 10.3389/froh.2023.1031574

**Published:** 2023-07-14

**Authors:** Carlos S. Smith

**Affiliations:** ^1^ Department of Dental Public Health and Policy, Virginia Commonwealth University School of Dentistry, Richmond, VA, United States; ^2^ Affiliate Faculty, Oral Health Equity Core, Institute for Inclusion, Inquiry and Innovation, Virginia Commonwealth University, Richmond, VA, United States

**Keywords:** ethics, clinical, social determinansts of health, structural determinants of health, ethical decision making (EDM), bias in health care

## Abstract

**Objectives:**

Clinical ethical decision-making models are largely geared toward individual clinician choices and individual patient outcomes, not necessarily accounting for larger systemic issues that affect optimal patient outcomes. The purpose of this paper is to provide an ethical decision-making model, drawing upon systems orientation and ethical theory, specifically incorporating and aiding in the mitigation of social and structural determinants of health.

**Methods:**

This paper presents a systems-oriented ethical decision-making framework derived from the analysis and critique of the Four-Box Method for Ethical Decision-Making by Jonsen, Siegler, and Winslade. Drawing upon both deontological and utilitarian ethical theory, the developed framework will assist providers, organizations, and health system leaders in navigating the increasingly complex ethical dimensions of care delivery for underserved patient populations, who are largely impacted by social and structural determinants of health.

**Results:**

The needs of underserved patients are inextricably linked to various social and structural determinants of health that, if left unaddressed, result in even poorer health outcomes, exacerbating existing health disparities. A systems-oriented ethical decision-making framework, centered on obligation, duty, and a utilitarian view of the optimal good, will aid providers, organizations, health system leaders, and community stakeholders in navigating the increasingly complex ethical dimensions of care delivery for underserved patient populations.

**Conclusion:**

Socioeconomic and political contexts have a significant impact on the way society is organized, how people are positioned in society, and how they will fare in terms of their health. Healthcare providers, including dentists, are largely unaware of and insensitive to the social issues that underlie the biological or psychological concerns that patients from socially disadvantaged backgrounds face. A systems-oriented ethical decision-making model will aid in mitigating social and structural determinants of health and the dental profession's obligations to the underserved.

## 1. Introduction

Social determinants of health (SDOH), as defined by the World Health Organization, are those conditions in which people are born, grow, work, live and age, and the wider set of forces and systems shaping the conditions of daily life (1). In short, the evolution of the SDOH concept came about from a need to be able to describe those influences on health outside of healthcare itself. Often, these SDOH play a significant role in who becomes sick, is riddled with disease, or lacks the opportunity to be the healthiest they could be. SDOH are estimated to account for more than 50%–60% of health outcomes (2), with some studies indicating that social determinants account for up to 80% of health outcomes (3). Oral disease is among the most significant unmet health needs in the world, and populations most prone to these diseases are also the most vulnerable: the poor, the very young, the elderly, those with disabilities, and those with comorbidities (4–6). While SDOH affect all patients, they are particularly important factors when looking to improve health outcomes among underserved populations.

Dental providers, especially those practicing in health professions shortage areas (including dental schools, federally qualified health centers (FQHCS), and other community-based clinical settings), are on the front lines serving individuals with complex dental, health and behavioral health needs, many of whom may lack insurance (7, 8) and experience high rates of substance use, interpersonal violence, homelessness, and unemployment (9–13). Moreover, dental pain is one of the top three drivers of costly and inefficient use of emergency department services, with recent studies estimating total ED oral health charges at more than $2.4 billion, with an average charge per dental visit of $992 ($994 for adults and $971 for children younger than 18) (14). Interventions for most patients presenting for dental pain inside EDs are minimal, and often, referral systems to dental providers are lacking at best.

The needs of underserved patients are inextricably linked to various social factors and SDOH; if left unaddressed, the result is even poorer health outcomes and exacerbation of existing health disparities (15). Healthy People 2030 has grouped SDOH into five domains: economic stability, education access and quality, health care access and quality, neighborhood and built environment, and social and community context (16). Examples of SDOH include: safe housing, transportation, and neighborhoods; racism, discrimination, and violence; education, job opportunities, and income; access to nutritious foods and physical activity opportunities; polluted air and water; as well as, language and literacy skills (16). Two things are clear: (a) promoting individual healthy choices will not eliminate the vast amount of disparity that exists across varying demographics of patient populations and (b) SDOH speak to larger systems issues that some scholars are defining as structural determinants of health. The purpose of this paper is to provide an ethical decision-making model, drawing upon systems orientation and ethical theory, specifically incorporating and aiding in the mitigation of social and structural determinants of health.

## 2. The social and the structural

The tension between social and structural determinants of health can be found in the very definition of SDOH provided by the WHO in the notion of social factors but also “the wider set of forces and systems shaping the conditions of daily life” (1). Those wider sets of forces speak of structural processes that determine the unequal distribution of these factors among groups resulting in social stratification of individuals within the socioeconomic and political contexts (17). Although not quantifiable on an individual level, these socioeconomic and political contexts have a significant impact on the way society is organized, how people are positioned in society, and how they will fare in terms of their health. This pushes against the notion that differences in oral health outcomes are frequently ascribed to personal socioeconomic and demographic traits (18). In order to properly address inequalities, structural variables that affect people' social, economic, and political surroundings must be recognized since they may facilitate or obstruct the adoption of healthy lifestyle choices. The degree of income disparity, labor market characteristics, health insurance coverage, public/private service delivery mix, accessibility to services, and the scope of inter-sectoral policies have all been identified as structural drivers of health outcomes and inequalities.

An example can be found in what may seem like a simple issue: access to fresh fruits and vegetables. Centering the social element—issues of grocery store access or proximity to grocery stores that actually offer fresh produce within their market—vs. corner stores, bodegas, or convenience stores that primarily populate underserved urban areas or rural areas that may lack any of the above. Social factors would also include transportation to access said grocery store and the economic ability to pay for one's groceries. A structural determinant lens would ask questions such as what policies (local, regional, state, and national) determine grocery store placement and success. Tax incentives for corporations, actualized and perceived violence or crime statistics, historic [and current] housing discrimination and who has access to mortgages, economic empowerment and wealth building strategies, livable wage or minimum wage policies, unionization of hourly wage earners, public transportation and environmental sustainability, and the list goes on. In connecting this example to health outcomes, the lack of access to fresh fruits and vegetables, a patient's lack of access to healthy foods in turn lends itself to poor, or less than ideal, nutrition. Poor nutrition then raises the risk of various health conditions, from increased caries and other oral health challenges to heart disease, diabetes, obesity, and more.

Particularly with the policy component, and the link to political structures, there have been recent movements surrounding political determinants of health. Political determinants of health are those that undergird multiple intersecting and interacting determinants of legal and political determinants, operating at every level and impacting the entire lifespan (19). Particularly when one considers large systemic and structural challenges such as racism, sexism, homophobia, ableism, xenophobia, transphobia, and the like, it becomes clear that these root causes of many inequities seen across healthcare delivery and systems must be addressed. In fact, many social and political structures and policies, both in the US and globally, were born out of racism, classism, and gender oppression (20). With scholars showing that structural (and political) determinants, such as the characteristics of oral health care systems, as well as social and economic conditions shape individual-level determinants and population-level oral health inequality, the inquiry of ethical sensibilities and responsibilities is a logical step. What are the ethical responsibilities of oral health professionals, both individually and collectively, to address social and structural determinants of health?

## 3. An ethical framing

Ethics has long been defined as a branch of philosophy and theology that involves systematizing, defending, and recommending concepts of right and wrong behavior. The American College of Dentists defines ethics as studying systematically what is right and good with respect to character and conduct (21). In short, ethics is about choices. In deciding whether or not to take action, oral health professionals and members of the oral health team (dental hygienists, dental therapists, dental assistants, and office personnel) must constantly study, consider, and resolve a variety of ethical concerns that are constantly changing (22). Ethics affect every decision made in the dental office and are inextricably linked to the daily decisions of overall dental practice. It is both an individual and a collective endeavor to strive to exemplify the highest standards of dental ethics and moral behavior.

What one dentist chooses to do, or not do, has implications and consequences not only for that individual but also for the profession as a whole (23). A previous dentist's choice to act, or not to act, has the potential to heavily influence a patient's view of both that dentist specifically, as well the patient's view of dentists generally and the profession as a whole. Studies have shown that an individual's dental health may be impacted by the abilities, dispositions, and philosophies of different dentists they may have experienced throughout their lives (24).

The Four-Box Method for Ethical Decision-Making by Jonsen, Siegler, and Winslade was analyzed and critiqued in this research to create a systems-oriented ethical decision-making framework. Ethical problems are analyzed in the context of four domains: medical indications, patient preferences, quality of life, and contextual features (i.e., social, economic, legal, and administrative) (25). Each topic can be approached through a set of specific questions with the goal of identifying the various circumstances of a given case and linking them to their underlying ethical principle (26). The developed framework will help providers, organizations, and health system leaders navigate the increasingly complex ethical dimensions of care delivery for underserved patient populations, who are significantly impacted by social and structural determinants of health. It draws on both deontological and utilitarian ethical theory.

Deontological ethical theory is based on individuals who uphold their obligations and duties when making moral decisions (27). This implies that a person will uphold his or her duties to another person or society since doing so is morally right. For example, someone imploring a deontological lens will always honor their commitments to friends and uphold the law. Given that their decisions are based on their predetermined obligations, those who follow the deontological theory will make choices that are quite consistent. Deontological principles are the ethics of obligation, according to which no harm is permitted even if it results in favorable outcomes. As a result, choices based on deontological ethics may be appropriate for an individual even though they may not benefit society as a whole (28).

In utilitarian ethics, outcomes justify the means or ways to achieve it, whereas in deontological ethics, duties/obligations are of prime importance (i.e., the end/outcomes may not justify the means) (29). The foundation of utilitarian ethical theories is one's capacity to foresee the results of one's actions. According to a utilitarian, the morally just decision is the one that benefits the greatest number of people (30). Act utilitarianism and rule utilitarianism are the two varieties of utilitarianism (31, 32). Act utilitarianism adheres strictly to the utilitarian definition: one does the things that help others the most, regardless of their own interests, emotions, or cultural restraints like laws. Fairness and consideration of the law are important to rule utilitarianism. A utilitarian looks for ways to help as many people as possible while still acting in a way that is fair and reasonable. Utilitarianism thus has the added advantage of valuing justice while also including beneficence. Deontological ethics are patient-centered by nature; as a result, ends do not justify means. However, utilitarian ethics, which lean more toward a focus on society, value concern for the greatest well being for the largest number of people; as a result, ends justify the means (31).

## 4. Ethics, bias and a systems approach

For many decades, dental ethics has primarily focused on professional codes of ethics, examining and often policing individual/group behavior, policies, practice, and compliance (33). Professionalism, with ethics at its foundation, concerns the behavior of a healthcare provider's duty to uphold the social contract (21). Professionalism extends ethics to include the conduct, aims, and qualities that characterize a professional or a profession. It can be further defined as an embodiment of positive habits of conduct, judgment, and perception on the part of individual professionals and professional organizations (21). Safety-net dental settings offer free or reduced cost care to low-income families, with government or grant funding offsetting expenses (33). As such, they are vulnerable to financial instability, irregular staffing, long wait times, and even closures, further limiting access to oral health care for vulnerable populations. Cost and availability are significant barriers to accessing dental care for low-income Americans. State Medicaid programs are an important component of the dental safety net and enable access to care by removing cost as a barrier (34). Yet, simply providing a form of public dental insurance does not ensure access. For patients to access care, dental professionals must be available in the community, enrolled in Medicaid programs, and willing to provide care to Medicaid recipients on an equitable basis as they do with private pay patients (34).

Studies have shown that healthcare providers, including dentists, are unaware of and insensitive to the social issues that underlie the biological or psychological concerns that patients from socially disadvantaged backgrounds face. Due in part to the dental office, including front-office and clinical staff, exhibiting bias and differential treatment of patients receiving social assistance, dentistry has openly shown discriminatory and differential behaviors (35–38). Unfortunately, discriminatory behavior within dentistry is not limited to the patient experience alone. Within dental education learning environments, students have reported discrimination, destructive communication, belittlement, and isolation (39). Moreover, in studies of Black, Hispanic, and American Indian/Alaska Native dentists, an overwhelming majority reported experiencing discrimination within both practice and dental school (40). Recent studies call for dental education and dentistry as a whole to fully commit to inclusive learning and practice environments and to specifically commit to anti-racist practices, policies, and procedures (41). WIth such robust evidence and practice experiences speaking to bias in working with underserved populations, both across the patient and practitioner spectrum, the dental professional and the profession as a whole must deliberately grapple with their perspective roles in mitigating issues around access to care, particularly as it relates to social and structural determinants of health.

Organizational ethics is concerned with the ethical responsibility of the organization as a whole to conduct its business and patient care practices in an honest, decent, and proper manner (42). In health care management and delivery, organizational ethics aims to understand and frame administrative and management ethical issues as opposed to clinical or professional ethics, case consultations, or clinical research. What are the ethical implications of organizational decisions and protocols of practices that those in health professions shortage areas or primarily treating underserved patient populations must face if they put the social contract front and center? What does it mean ethically and professionally for an organization and those individuals working within it to see themselves as dental safety nets? How do they ethically, as both an organization and a collection of individuals, carry out an organizational and ethical identity that fulfills the healthcare professional social contract?

Studies report that dentists are more likely to participate in Medicaid if they are from a racial or ethnic minority group and/or practice pediatric dentistry (43–45). Studies have also shown that some oral health care providers do not accept Medicaid, or limit the number of Medicaid patients, because of administrative overhead and/or inadequate reimbursement for treatment (46). Dental schools, FQHCs, and other community based clinical settings, must begin to examine the bioethical issues, including and beyond Medicaid coverage, that present barriers to access to care and fulfillment of the social contract, such as citizenship, language, and dental distrust (46, 47). Studies have called into question the level of confidence a patient may have in the care provided by a hospital vs. a dentist, which points to the importance of trust in oral health care providers as a factor affecting Medicaid dental visits (46). Oral health practitioners, including students in school and dentists in practice, must examine their attitudes and practice beliefs concerning their willingness to serve the needs of under/unserved populations (48). Studies have shown that faculty interactions play an important role in students' attitudes about treating underserved patients (49).

More specifically, the role of role modeling, a central tenant within professionalism, and how dentists and dental students become acclimated to and form their professional identities must not be overlooked. What behavior is modeled for new associates brought into a practice with a focus on meeting the needs of underserved patients? Treatment of patients, a lack of empathy, and a misunderstanding of patient economic situations, all speak to bias that may exist within professional ranks. Particularly, given the vast disparities and inequities throughout global healthcare systems and practices, should professionalism definitions be extended to include disruption or dismantling of inequitable systems and organizational structures? While professionalism has long been defined as the embodiment of positive habits of conduct, judgment, and perception on the part of individual professionals and professional organizations (7). The redefining of professionalism and professional ethics must address societal challenges such as health injustices and inequality in light of the COVID-19 epidemic, the worldwide racial crisis around Black Lives Matter, and anti-Asian rallies and advocacy. The key to that reframe is an expansion of previous definitions to include intervention tactics, institutions, and practices—not only refraining from harm but actively interfering or taking action if wrong is being observed—rather than just abstaining from damage (10, 35). Academic health centers are starting to provide bystander intervention training in an effort to counteract and reduce impoliteness, discrimination, and biased behavior, taking on responsibility for providing safe learning, teaching, and practice environments (50–52). Thus, the development of a systems oriented ethical decision making framework to mitigate social and structural determinants of health is both a logical and innovative step.

## 5. Ethical decision-making models

Ethical decision-making for dentists can be relatively straightforward and simple or can delve into quite a complex process of weighing out options and various stakeholder viewpoints. Due to the ever-evolving complexity of dentistry and dental practice, several models of ethical decision-making have been developed and utilized over time. Most models contain several stakeholder issues and ethical principles for reflection (21). Professions, including dentistry, are largely defined as such in part because of self-governed and developed codes of ethics. A code of ethics defines the moral boundaries within which professional services may be ethically provided. Many dental organizations have codes of ethical conduct for guidance of dentists in their practice. The American Dental Association (ADA) has five guiding and fundamental principles, which are: patient autonomy, non-maleficence, beneficence, justice, and veracity (24).

Many models and frameworks exist to aid health care practitioners in managing ethical challenges that arise during clinical care. The most traditional perspective on dental ethics and moral decision-making comes from Ozar's Central Values of Dental Practice, a classic work. These values include: (a) the patient's life and general health; (b) the patient's oral health; (c) the patient's autonomy; (d) the dentist's preferred patterns of practice; (e) esthetic values; and (f) efficiency in the use of resources (24). One of the most recent developments in dental ethics has been the use of narrative ethics as a model for ethical decision-making. Narrative ethics enables one to deconstruct cases in a broader sense, with the ethical choices made more easily subject to reflection and evaluation (53). It also helps one think about an ethical scenario as a story and helps to better empathize with others' thoughts and feelings, enabling more thoughtful decision-making. Some criticism put forth concerning narrative ethics has focused on the lack of appeal to rules, principles, or other ethical constructs (54).

**Figure 1:**
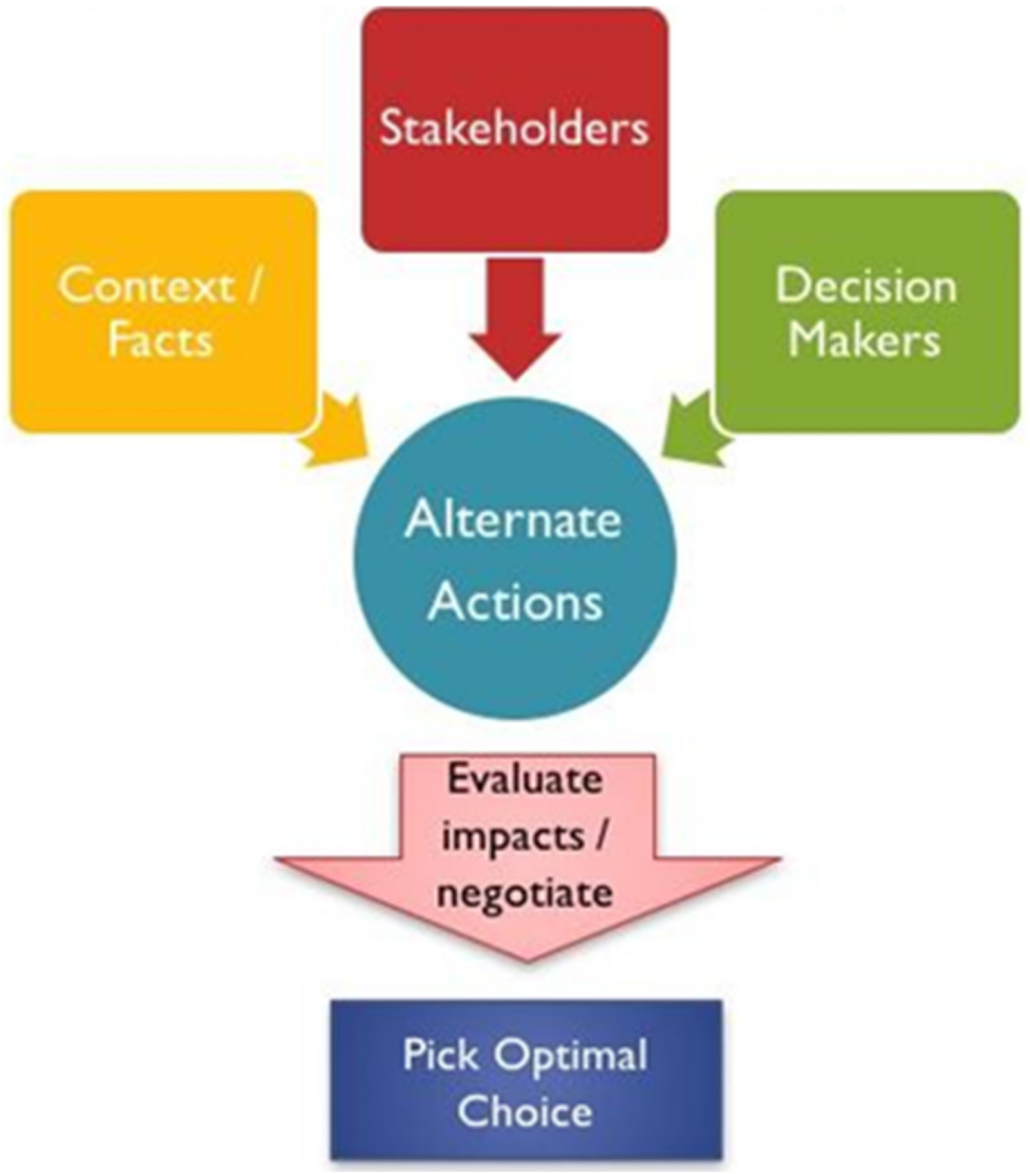
Narrative ethics.

**Figure 2:**
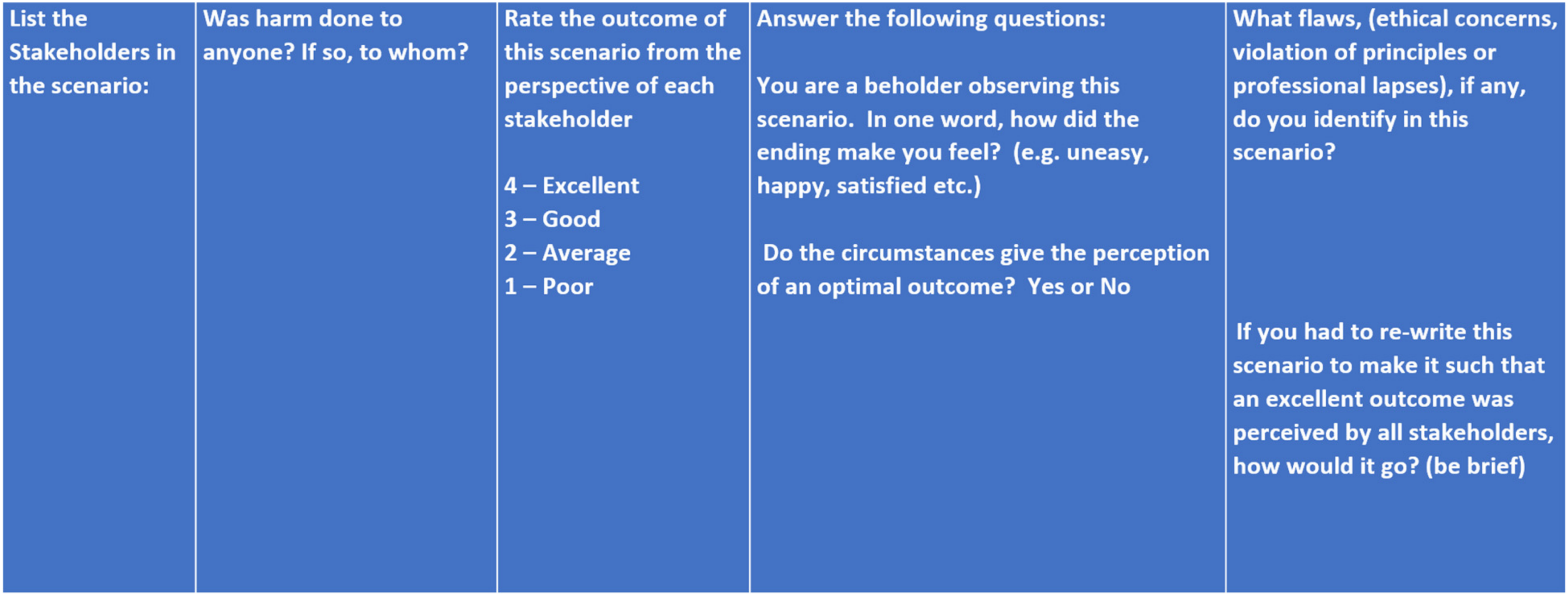
Roucka/more narrative ethics rubric.

Roucka and More have developed a specific narrative dental ethics decision-making model rubric and framework relying on both narrative and story as well as incorporating consideration of classic health care ethical principles. (Figures 1, 2) Their model includes: identifying the stakeholders; asking if harm was done to anyone and by whom; rating (4 being excellent and 1 being poor) the outcome from the perspective of each stakeholder; an inquiry into how the story makes one feel; determining if circumstances give the perception of an optimal outcome; identifying flaws (breach of principles, procedural, and/or ethical); and lastly, an attempt at rewriting the story to make the scenario such that an optimal outcome is perceived by all stakeholders (55). The narrative dental ethical decision-making approach promotes self-reflection, remembering through emotional connection, and aids in illuminating multiple points of view. It also enables the development of empathy (23).

## 6. Development of a systems oriented ethical decision making framework

The four-box model has previously been adapted to dentistry and dental education, providing further clarification within the four boxes and additionally connecting each to an ethical principle(s) adopted by the dental profession: patient autonomy, nonmaleficence, beneficence, justice, and veracity (56). Developed and adapted with the aim of restoring public trust in the dental profession, the adapted four-box model, A Case-Based Approach to Ethical Decision-Making, is a particularly useful tool because of its emphasis on team-based learning (TBL) modules. Such modules, formed around a “duty-specific” theme, can be created in an effort to overcome challenges frequently faced by the dental profession (access to care, the opioid epidemic, informed consent in the cognitively impaired), and they may well strengthen ethical consistency in the profession itself while also benefiting public trust and professional reputation at large (56).

**Figure 3:**
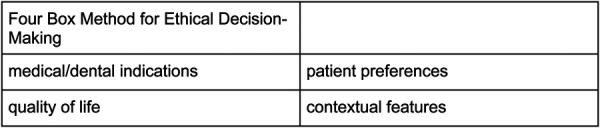
Four-box method for ethical decision-making. Jonsen AR, Siegler M, Winslade WJ. Clinical Ethics: A Practical Approach to Ethical Decisions in Clinical Medicine. 6th ed. New York, NY: McGraw-Hill; 2006.

**Figure 4:**
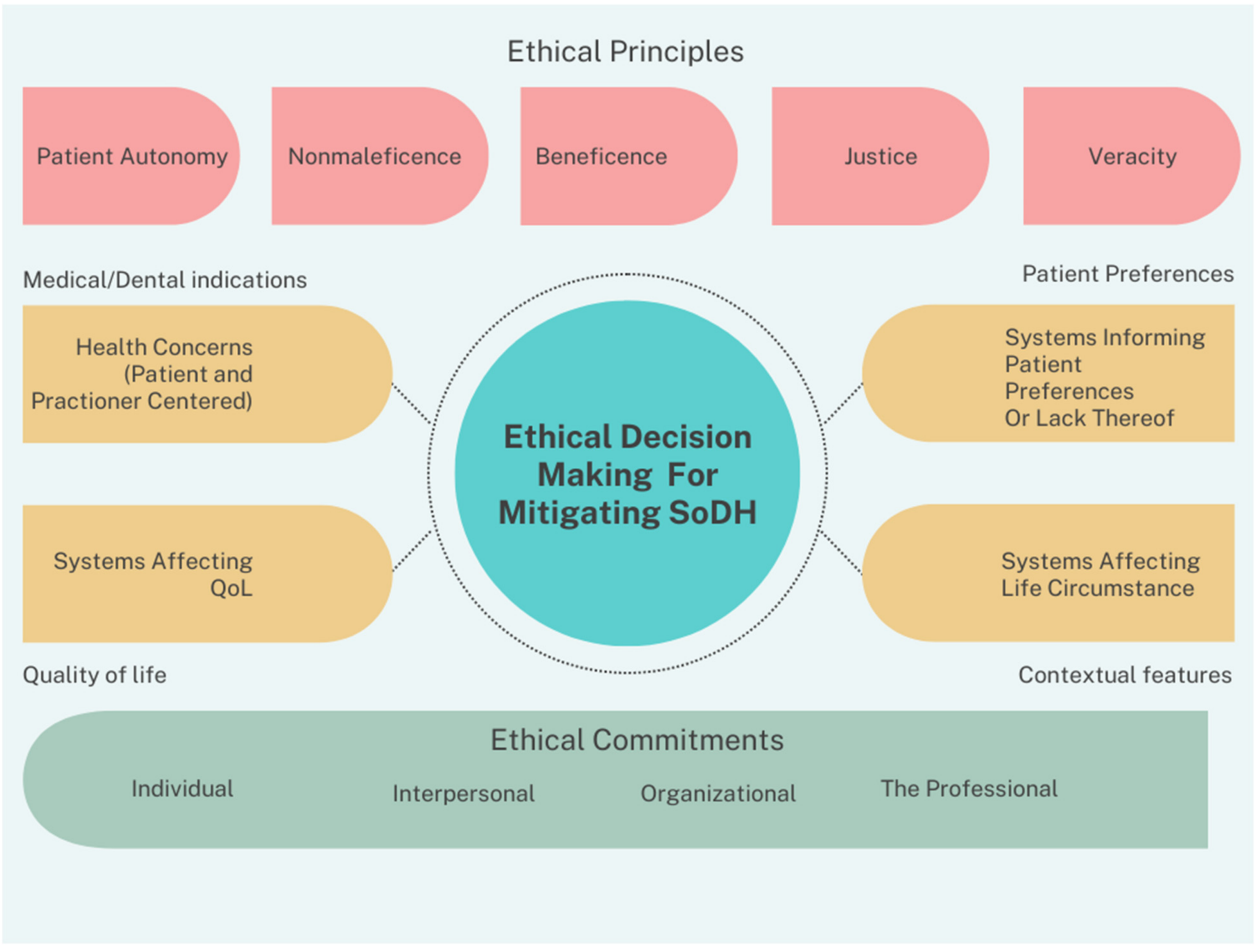
A systems oriented ethical decision making framework for mitigating social and structural determinants of health.

The adaptation of the Four Box Model (Figure 3), specific to dentistry, is particularly useful to the individual clinician and the profession as a whole. However, as addressing social and structural determinants clearly shows, the individual approach, while beneficial, does not alone paint the entire proverbial picture. Moving towards solutions-oriented outcomes, a Four Box Model that speaks to systems thinking and incorporates social and structural determinants directly would be helpful. As can be seen in Figure 4, a systems-oriented ethical decision-making framework would incorporate the five key principles of dental ethics as overarching considerations while also requiring the centering of key ethical commitments. The systems-oriented ethical decision-making framework draws on both deontological and utilitarian ethical theories. Ethical commitments are a central tenet of the multi-use ability of the framework, calling for critical reasoning concerning the individual, the interpersonal, the organizational, and the professional (the profession as a whole). The adaptation of the traditional Four Box Model to systems thinking expands the four domains of medical indications, patient preferences, quality of life, and contextual features to health concerns (including but not limited to both dental and medical issues) that are both patient and practitioner-centered, systems informing patient preferences or lack thereof, systems affecting patient quality of life, and systems affecting life circumstances. The use of this framework implores one to remain mindful that no single domain has more bearing or weight on the ethical decision-making process, but that each domain is given heavy and thoughtful consideration. Another benefit of the adapted four box model is its set of specific questions for consideration when faced with an ethical decision. Boxes 1–4 show how these questions can be expanded to include a systems thinking approach to both social and structural health determinants. The strengths of a systems approach is due to the fact that, in addition to interpersonal factors, organizational structures are needed to support providers in providing equitable care, such as infrastructure that facilitates training and resources needed to engage patients in shared decision-making and address social determinants of health (57).

Box 1 **Health concerns (Patient and Practitioner Centered)**

•  What is the patient's dental/medical problem? History? Diagnosis? Prognosis? Are there organizational protocols or interventions that may aid in diagnosis or health management, such as BP, HbA1C, vaccination administration, or health screening?
•  Is the problem acute? Chronic? Critical? Emergent? Reversible?
•  What are the patient's goals of treatment? What are the provider's goals of treatment? Are there organizational goals that contradict the patient's or provider's goals?
•  What are the probabilities of success of each treatment option? Are there circumstances where dental treatment won't be probable? If treatment, not probable, what is the monitoring or re-evaluation plan?
•  What are the plans in case of therapeutic failure?
•  In sum, how can this patient be benefited by oral healthcare care, and how can harm be avoided?

Box 2 **Systems informing patient preferences or lack thereof**

•  Is the patient mentally capable and legally competent? Is there evidence of capacity? A systems view would ask what tools of capacity assessment are available and then require the implementation of using those tools chairside.
•  If competent, what is the patient stating about preferences for treatment?
•  Has the patient been informed of the benefits and risks, understood this information, and given consent? Is there a need to assess the oral health literacy of the patient or the community from which the patient arrives? Are there issues related to SDOH in terms of patient education level and employment history?
•  If incapacitated, who is the appropriate surrogate? Is assent able to be obtained if consent is not able to be obtained? Is the surrogate using appropriate standards for decision making? Who has access to surrogate information and how do surrogates, healthcare powers of attorney, etc. work? Are there interventions the organization or profession can provide?
•  Has the patient expressed prior preferences?
•  Is the patient unwilling or unable to cooperate with medical treatment? If so, why?

Box 3 **Systems affecting quality of life.**

•  What are the prospects, with or without treatment, for a return to normal life?
•  What physical, mental, and social deficits is the patient likely to experience if treatment succeeds?
•  Are there biases that might prejudice the provider's evaluation of the patient's quality of life? Have providers undergone implicit bias, cultural humility, and emotional intelligence training to mitigate bias?
•  Is the patient's present or future condition such that his or her continued life might be judged as undesirable? What process is used for making such a judgment? Who is involved in such a judgment?
•  Is there any plan and rationale to forgo treatment?
•  Are there plans for comfort and palliative care? If a patient is in hospice or facing end-of-life care, how is oral comfort incorporated vs. being overlooked? What system resources are available to include oral health related QOL in end-of-life care and hospice?

Box 4 **Systems affecting life circumstances**

•  Are there family issues that might influence treatment decisions? How does the SDOH of family and community support aid or complicate treatment decision making?
•  Are there provider issues that might influence treatment decisions? Systems issues such as office hours, availability of appointments, and the number of treatment visits needed to complete treatment?
•  Are there financial and economic factors? Are there problems with the allocation of resources?
•  Are there religious or cultural factors? Are language interpreter services available, adequate, and in use?
•  Are there limits on confidentiality? Is there adequate wifi in the patient's home region for telehealth screening or interventions? How accessible is the electronic health record (EHR) or patient portal for the patient or surrogate?
•  Are there issues of public health and safety that affect treatment decisions?

### 6.1. An example scenario

Despite having a strong theoretical foundation, the framework's design aims to be simple to use and execute. Similar to decision trees, the framework implores the user to ask several questions of themselves, as can be seen in Boxes 1–4. A scenario may unfold this way: *you are the managing or lead dentist at a FQHC in an urban setting with both large Spanish-speaking and Somalian immigrant communities that approximate your clinical site. Lately, no-shows, same day cancellations, and lack of treatment plan acceptance have been frustrating staff, clogging phone lines, and suggestions around common, yet ethically questionable practices of double booking patients dependent on patient form of payment (cash pay, private insurance vs. public insurance) are already being implemented, albeit with little success.* How would you and the FQHC leadership attempt to deal with this ethical challenge in practical terms? Using the system-oriented ethical decision-making framework, one can begin to interrogate not only individual needs and choices but also how systems impact said individual outcomes.

Figure 4 allows for the consideration of both classic ethical principles (patient autonomy, nonmaleficence, beneficence, justice, and veracity) and ethical commitments across stakeholder groups (individual, interpersonal, organizational, and professional). One solution would be to gather focus groups of patients for direct engagement with their expressed needs and concerns. Accounting for medical/dental indications, are there ways in which patient medical and dental needs can be simultaneously met? Particularly in the FQHC context, is there interprofessional delivery of care, a shared electronic health record (EHR), or easy means of communication amongst providers, etc.? If not, then should strategies and policies change to implement an interprofessional approach? Taking account of both patient and practitioner needs, one can move through the framework to patient preferences that are impacted by systems; examples may include transportation to appointments; clinic hours and desire for extended evening, early morning, or weekend appointments; referrals for child care coverage or perhaps an organizational grant application to provide childcare on site; language and interpretation services; etc. Moving towards systems affecting quality of life, what is life like for the patient if they don't get the healthcare they need? Is there a need for implicit bias training and deescalation training for staff around patient interactions or the perceived bias of patient circumstances? And finally, considering those systems that affect life circumstances, if transportation is an issue, what is the closest available public transportation option? Even in those locales where public insurance provides transportation vouchers or supplementation—is there a bias or mistreatment of those patients in those settings? If language interpretation services are virtual or technologically based, what quality control measures are in place to evaluate how patients respond to or feel about said services?

A systems-oriented ethical decision-making framework also reminds the user that ethics and ethical decision-making are not conducted in a vacuum. Dentists contribute a range of perspectives and life experiences to the ethical decision-making process. This would encompass a variety of factors, including but not limited to: one's background and upbringing, religious convictions or lack thereof, educational background, professional experiences, setting of practice, expectations of the patients, social norms, and more. These various life experiences and perspectives shape dentists' understanding and wellbeing, ultimately affecting patient outcomes. Undoubtedly, free discussion and deliberate consideration of ethical concerns result in better-quality decisions made by providers. Ultimately, this will yield a better life for patients and increased satisfaction and altruism for dentists and the dental care team. Limitations of this work may include the heavy theoretical framing of the work, which doesn't always lend itself to practicality and immediate application based usage; however, with systems views in mind, institutional, organizational, educational, corporate, and practice leaders will do well to implement its use.

## 7. Conclusion and next steps

In addition to ethical decision-making, there is room for improvement in understanding SDOH across the dental professional landscape, from education for active clinicians within and outside of safety-net clinical settings to dental school curriculum or even mandated board licensure renewal requirements for specified SDOH continuing education. SDOH and structural determinants of health are, without a doubt, ethical issues across the oral health professions and dental teams. Although primary care medicine has begun implementation of SDOH screening protocols in recent years, dental practices, most commonly, do not include assessments of social determinants in their intake processes, new patient exams, or when establishing patient medical histories. Thus, it is of dire importance for health care providers of all disciplines, including oral health, to screen patients for social factors that may affect their health (58). To exacerbate matters, dental schools, to a large extent, are not training providers to assess and address social determinants of oral health, or if they are, the teaching is largely regulated to didactic or simulation instruction only, perpetuating disparities in access to dental care and poor dental health outcomes among historically vulnerable and marginalized communities (59).

With the building blocks of both deontological and utilitarian ethical theory, those involved in making health care, health system, and health policy decisions should have a propensity for empathy and think about issues from a variety of ethical angles and professional obligations. The conceptualization and use of an ethical decision-making framework that specifically factors in social and structural determinants of health, presents a clear opportunity for resolving moral and ethical challenges while promoting greater beneficence and justice for patients and all parties involved. A systems-oriented ethical decision-making framework enables clinicians and teams who treat and serve underserved patients to more adequately consider the ethical dimensions of care, how they intersect with clinical care, and how deliberate attention to them may improve optimal care and health for all.

## Data Availability

The original contributions presented in the study are included in the article, further inquiries can be directed to the corresponding author.
